# The purposes of research support: the Australian example

**DOI:** 10.1007/s12551-023-01043-y

**Published:** 2023-02-02

**Authors:** Ronald J. Clarke

**Affiliations:** School of Chemistry, University of Sydney, Sydney, NSW 2006 Australia

**Keywords:** Research funding, Political interference, Fundamental versus applied research

**Fig. 1:**
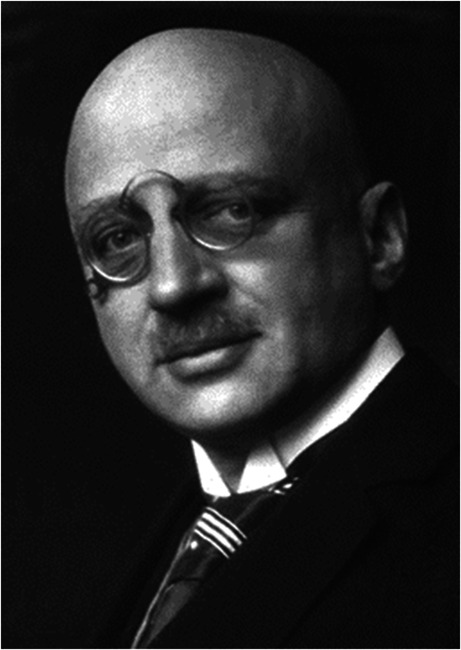
Fritz Haber ca. 1919, Research in service of humanity or the nation. https://en.wikipedia.org/wiki/Fritz_Haber

Why should scientific research be supported with public money? There are several possible answers depending upon who is asked. In his farewell letter to his colleagues at the Kaiser-Wilhelm-Institute of Physical Chemistry and Electrochemistry (now the Fritz-Haber-Institute) in Berlin-Dahlem, Fritz Haber (see Fig. 1), the famous German physical chemist and Nobel prize winner, wrote in 1933 that for the past 22 years, he had devoted himself to serving humanity in times of peace and to serving the Fatherland during wartime (Kaiser, 635:210–220, 2002; Chmiel et al., 1986). No doubt, Fritz Haber would also have been of the opinion that research support from the Government should serve either humanity or the nation. But politicians sometimes see things from a different perspective, and often a very short-term one. If decisions regarding the selection of projects to be supported are left to politicians, the danger exists that science could be politically abused. This can particularly be the case for applied research, where results are much easier for the public to understand than in the case of more abstract fundamental research. If politicians can say before the end of an electoral term that they have supported research that has outcomes that can clearly be seen by the public to be positive, e.g., the successful treatment of a disease, an increase in agricultural harvest or a more environmentally acceptable method of ore extraction, then that improves their re-election chances. For this reason, in many countries, including Germany, politicians are excluded from decisions on research support, or their influence on the decisions is severely limited via the participation of scientific experts. But what is the situation in my home country, Australia?

On Christmas Eve 2021, six applications for research support which had already been approved for funding by the Australian Research Council (ARC) were rejected by the Education Minister, Stuart Robert. The Minister made use of his veto right. All of these applications were in the field of the Humanities or Social Sciences, but applications in the natural sciences could just have easily met with the same fate. The title of one of the rejected applications was: “New Possibilities: Student Climate Action and Democratic Renewal.” For a Government like that of the then Prime Minister Scott Morrison, which was known for its hesitant reaction to climate change, research on student protests to climate change was perhaps not politically desirable. However, as a justification for the veto the Minister merely stated that the six rejected applications did not represent good value for money. In March 2022, a review of the Minister’s actions was held in Parliament in Canberra. Senator Carr of the Labor Party, which at that time was in opposition, asked what criteria the Minister had applied in determining value for money. The representative of the Government could not offer any answer. Senator Carr suggested as a possible explanation that the Minister had “perhaps got out of the wrong side of the bed that morning.”

Regardless of whether a project is rejected for political reasons or for other reasons, the decision should not be left in the hands of a single person. As in Germany, in Australia, all applications are first reviewed by experts in their respective fields. After that, a ranking of the applications is produced by so-called “ARC Colleges of Experts.” Based on the ranking and the amount of money available, decisions are made regarding which applications should be funded. If at the end of this process, lasting several months, the Minister rejects grant approvals, then that represents a contempt of the subject experts. After the veto, two of those experts even tendered their resignations as a consequence. Professor Duncan Ivison, Deputy Vice-Chancellor of the University of Sydney, compared the rejection of the proposals by the Minister with selection of the Australian Olympic Team by the Sports Minister. As one would expect, the topics of the projects are highly complex and specialized so that no one person could be in a position to judge the value of applications across all fields of the natural sciences, humanities, and social sciences. Furthermore, not only is political interference in the grant approval process contempt of the reviewers; even worse, it damages the international reputation of a country. Who overseas would want to collaborate with scientists whose research funding could be cut by the decision of a politician?

A further important question, which interests politicians particularly, is the question of the distribution of public money between fundamental and applied research. Every Government naturally has a justified wish to obtain the greatest possible profit from the invested money. But how does one obtain the greatest possible profit? From the actions of the Australian Education Minister, his opinion can be clearly discerned. In an open letter to the CEO of the ARC, Professor Sue Thomas, on the 6th of December 2021, the Minister declared his expectation that the ARC should be immediately reformed so that research is favored in fields chosen by the Government and which the Government believes are in the national interest. These fields include especially the production of commodities for sale overseas to improve the gross domestic product. A few days after the release of the Minister’s letter, Professor Thomas resigned from her office as CEO of the ARC.

How does this state of affairs compare with that in Germany? The decisive point is: How is public money best invested in order to obtain the most effective results? In Germany, luckily, there are several examples to show that the value of fundamental research, unencumbered by political influence, is recognized by Governments of all political persuasions. Prime examples are the Max-Planck-Society and the Alexander-von-Humboldt-Foundation, which are both based on the fundamental principle of funding excellent researchers instead of projects, so that the researchers have the freedom to follow their passions and curiosity (Schwarz 2017; Aufderheide 2022). The scientific freedom and financial support that employees of the Max-Planck-Institutes enjoy are the envy of scientists the world over. To obtain important results, often many years are required, mostly much longer than an electoral period. A Government must, therefore, exercise much patience and wait for its investments to bear fruit. For example, as the Secretary-General of the Humboldt Foundation, Dr. Enno Aufderheide, recently explained (Aufderheide 2022): The basis of modern medical MRI-investigations goes back to the fundamental discoveries of spin quantization by Otto Stern and Ernst Gerlach in the year 1920. Governments must, therefore, give up any hope of gaining political capital from investment in fundamental research. However, in the long term, the possible eventual gains through investment in fundamental research may well trump those obtained by investment in applied research. A Government must be prepared to take a risk, because great discoveries through fundamental research are never guaranteed. Researchers are like gold prospectors. Not every prospector strikes gold, but considering the potential gains, the hard work is worth it. The best strategy for research support is thus to be prepared to take risks and to fund fundamental research. While on this topic it is interesting to note that even in the case of successful researchers not every project is a success. An unsuccessful project of Fritz Haber was his search for gold in seawater in the 1920s, in the hope that thereby he could alleviate his country of the high reparation payments to France after the First World War. As a side note to this, Fritz Haber’s expectation that the extraction of gold from seawater could be profitable was based on an overestimation from an Australian chemist from the University of Sydney, Professor Archibald Liversidge (Liversidge 1895). Even in science, you cannot believe everything that you read.

In conclusion, up until recently in Australia, we had a Government which treated academic researchers with contempt and wanted to support applied research at the expense of fundamental research in order to obtain short-term national and political advantage. On the 21st of May 2022, however, everything changed. On this day, a federal election took place. The governing Liberal Party of Prime Minister Scott Morrison was defeated and the Labor Party under Anthony Albanese formed the new Government. In addition, the Greens and a large number of independent candidates, who want more action against climate change, won many seats. The Labor Party has promised to do more for universities and to restructure the system of research funding so that political interference in funding decisions is no longer possible. That still remains to be seen, but Australian researchers now at least have the hope that positive changes are on the way.
